# White Blood Cell Scintigraphy for Fracture-Related Infection: Is Semiquantitative Analysis of Equivocal Scans Accurate?

**DOI:** 10.3390/diagnostics11122227

**Published:** 2021-11-29

**Authors:** Paul Bosch, Frank F.A. IJpma, Geertje A.M. Govaert, Inge H.F. Reininga, Jean-Paul P.M. de Vries, Andor W. J. M. Glaudemans

**Affiliations:** 1 Department of Surgery, University of Groningen, University Medical Center Groningen, Hanzeplein 1, 9713 GZ Groningen, The Netherlands; f.f.a.ijpma@umcg.nl (F.F.A.I.); i.h.f.reininga@umcg.nl (I.H.F.R.); j.p.p.m.de.vries@umcg.nl (J.-P.P.M.d.V.); 2 Department of Trauma Surgery, University of Utrecht, University Medical Center Utrecht, Heidelberglaan 100, 3584 CX Utrecht, The Netherlands; g.a.m.govaert@umcutrecht.nl; 3 Department of Nuclear Medicine and Molecular Imaging, University of Groningen, University Medical Center Groningen, Hanzeplein 1, 9713 GZ Groningen, The Netherlands; a.w.j.m.glaudemans@umcg.nl

**Keywords:** WBC scintigraphy, fracture-related infection, FRI, semiquantitative analysis, infection, imaging

## Abstract

Purpose: White blood cell (WBC) scintigraphy is considered the gold-standard nuclear imaging technique for diagnosing fracture-related infection (FRI). Correct interpretation of WBC scans in FRI is important since a false positive or false negative diagnosis has major consequences for the patient in terms of clinical decision-making. The European Association of Nuclear Medicine (EANM) guideline for correct analysis and interpretation of WBC scans recommends semiquantitative analysis of visually equivocal scans. Therefore, this study aims to assess the diagnostic accuracy of semiquantitative analysis of visually equivocal WBC scans for diagnosing FRI. Methods: A retrospective single-center study was performed in consecutive patients who received WBC scintigraphy in the diagnostic work-up for FRI between February 2012 and January 2017. All the visually equivocal scans were analysed using semiquantitative analysis by comparing leukocyte uptake in the manually selected suspected infection focus with the contralateral bone marrow (L/R ratio). Cut-off points for a ‘positive’ scan result of >0%, >10% and >20% leukocyte increase between the early and late scans were used in separate analyses. The discriminative ability was quantified by calculating the sensitivity, specificity and diagnostic accuracy. Results: In total, 153 WBC scans were eligible for inclusion. After visual assessment of all the scans, 28 visually equivocal scans were included. Dichotomization of the ratios using the cut-off of >0% resulted in a sensitivity of 30%, a specificity of 45% and a diagnostic accuracy of 40%. The >10% cut-off point resulted in a sensitivity of 18%, a specificity of 82% and a diagnostic accuracy of 66%. The >20% cut-off point resulted in a sensitivity of 0%, a specificity of 89% and a diagnostic accuracy of 67%. Conclusion: Semiquantitative analysis of visually equivocal WBC scans is insufficient for correctly diagnosing FRI.

## 1. Introduction

Diagnosing fracture-related infection (FRI) can be challenging, and several diagnostic imaging modalities have been proposed [1,2,3,4,5,6,7]. White blood cell (WBC) scintigraphy has been proven to be accurate in diagnosing infections of both bone and soft tissue. A recent retrospective study from our study group analyzed the accuracy of WBC scintigraphy in 192 patients with suspected FRI using the current acquisition and interpretation guidelines and found a diagnostic accuracy of 92% using qualitative (visual) assessment [6]. Results of other studies are difficult to compare due to substantial heterogeneity in protocols for image acquisition and interpretation [2,4].

In 2013, a dual timepoint acquisition protocol was proposed with imaging 3–4 and 20–24 h after injection of autologous technetium-labeled white blood cells. This protocol has become the standard and is also recommended in the latest European Association of Nuclear Medicine (EANM) guideline on WBC scintigraphy [8]. Increase in leucocyte uptake with time in intensity or size is considered to indicate an infection. In case of equivocal visual findings, semiquantitative analysis of leucocyte uptake between the early and late scans is recommend in the guideline. This semiquantitative method comparing the uptake in a manually selected suspected infection focus with the contralateral bone marrow showed high diagnostic accuracy in a small number of patients with FRI [7]. Similar results were reported in a group of suspected prosthetic joint infections [9].

Although promising, this semiquantitative method has never been assessed in a large cohort of patients with suspected FRI in accordance with the current AO/EBJIS consensus definition [10]. This is important because the result of this measurement is decisive in diagnosing FRI and its consequent treatment. False positive results may lead to overtreatment such as unnecessary surgical procedures and/or unjustified antibiotic therapy. False negative results may lead to delay in diagnosis and worsening of the disease. Therefore, the aim of this study was to assess the accuracy of semiquantitative analysis of visually equivocal WBC scintigraphy scans in patients with suspected FRI.

## 2. Materials and Methods

### 2.1. Study Design

This is a retrospective single-center diagnostic imaging study performed at University Medical Center Groningen (UMCG), a level I trauma center in the Netherlands. 

### 2.2. Patients

All the patients who underwent WBC scintigraphy for suspected FRI at UMCG between February 2012 and January 2017 with an equivocal result were eligible for inclusion. Scans were classified as equivocal by visual assessment using the following characteristics from the EANM guideline: similar or slightly decreasing (with time) uptake in the regions of interest (intensity or size); increase in bone marrow activity with time that interferes with the evaluation of suspected regions; slight increase in size over time without an increase in intensity of uptake; slight increase in size and/or intensity over time in the region of interest but with a similar increase over time in bone marrow activity; activity in the region of interest that remains unmodified with time (in size and/or intensity) with bone marrow activity that increases or decreases over time [8]. Excluded were patients for whom no diagnostic reference standard or minimal follow-up of 1 year was available.

### 2.3. Index Test

#### 2.3.1. Scan Technique

The index test consisted of white blood cell scintigraphy using [99m]Tc-HMPAO-labeled autologous leukocytes performed according to the current EANM guideline for the acquisition and interpretation of WBC scintigraphy [10]. Autologous WBC (mixed leukocytes) were radiolabeled with [99m]Tc-HMPAO and dual timepoint acquisition was used, with static images acquired 3–4 h and 20–24 h after an intravenous injection of 370–550 MBq [99m]Tc-HMPAO-labeled WBC. All images were acquired with decay-corrected acquisition times in accordance with the current EANM guidelines. Acquisition time for the first scan (2–4 h) was 150 s per view in case of the dose of 370–550 MBq and 200 s per view for the dose of 250–370 MBq. Acquisition time for the second scan (20–24 h) was determined in relation to the time passed since the first scan. The acquisition times for the [99m]Tc decay correction can be found in Table 1. Subsequently, the images were displayed and analyzed using a total counts intensity scale with the same intensity threshold, thus avoiding observer bias. A SPECT (40 s per frame, 360° rotation) with a low-dose CT scan was performed for determining the exact anatomical location in all the patients in whom uptake was seen in the 4 h images. A SPECT/CT gamma camera system (Symbia T, Siemens Medical Systems, Knoxville, TN, USA) was used for all the scans.

#### 2.3.2. Image Interpretation

All WBC scintigraphy performed in the study period for diagnosing or excluding FRI was visually assessed by two experienced nuclear medicine physicians. Scans that were labeled visually equivocal were included in this study. All the equivocal scans were then assessed using the semiquantitative method; the reviewing nuclear medicine physicians were blinded for the clinical outcome.

**Figure 1:**
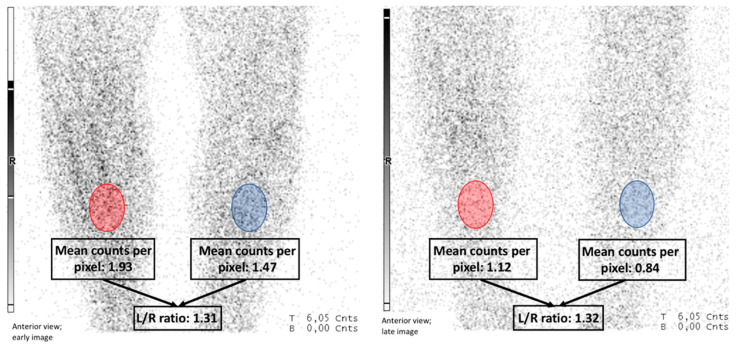
Example of L/R ratio calculation using semiquantitative analysis. L/R ratio calculation for the early (**left image**) and late (**right image**) scans of suspected FRI of a right tibial fracture. Red indicates the area of interest. Blue indicates the contralateral reference tissue. The mean counts per pixel were calculated within the selected area and the ratio was calculated in both the early and the late scans. In this case, the ratio remained stable between the early (L/R ratio of 1.3) and late (L/R ratio of 1.3) phases, which is considered negative for infection.

Semiquantitative analysis was performed by drawing a region of interest (ROI) over the suspected infectious focus and an automatically mirrored ROI over the contralateral reference tissue in accordance with the EANM guidelines [8]. If no clear region of interest could be selected based on WBC uptake, the area of interest was selected based on plain radiography of the initial fracture site and implant. The mean counts per pixel in these ROIs were recorded and the lesion-to-reference (L/R) ratio was calculated for both images. When the L/R ratios decreased or remained stable between the early (3–4 h) and the late (20–24 h) scans, the scan was considered negative for infection. When the L/R ratios increased in time, the scan was considered positive for infection. Hence, only the planar images were used for declaring a scan positive or negative for infection. An example is given in Figure 1. All semiquantitative analyses were performed by P.B. and verified by A.G. using the symbia.net software (Siemens, Knoxville, TN, USA). An example of an L/R ratio calculation is displayed in Figure 1.

### 2.4. Reference Test

The reference test for FRI consisted of the presence of confirmative criteria for FRI according to the AO/EBJIS FRI consensus definition during the follow-up period [11]. These criteria are the presence of fistulae, sinus or wound breakdown, purulent drainage or pus. In surgically treated cases, FRI is confirmed when (1) there are at least two out of six deep tissue cultures with identical pathogens, (2) the presence of microorganisms in deep tissue specimens confirmed by histopathological examination.

### 2.5. Statistical Analysis

All the calculated ratios were dichotomized thrice using different cut-off points for a ‘positive’ scan result. First, the ratios were dichotomized by labeling the scans with decreased or comparative uptake as ‘negative’ and the scans with any increase in uptake (e.g., >0% increase) as ‘positive’. The same was done using a cut-off point of ≥ 10% increase and ≥20% increase, respectively, over time for a ‘positive’ scan result.

True positive (TP), true negative (TN), false positive (FP) and false negative (FN) results were described and displayed in contingency tables. Sensitivity, specificity and diagnostic accuracy were calculated per cut-off point.

Second, ratios were used as continuous values to calculate the area under the receiver operating characteristic (AUROC) as a measure of discriminative performance.

## 3. Results

**Figure 2:**
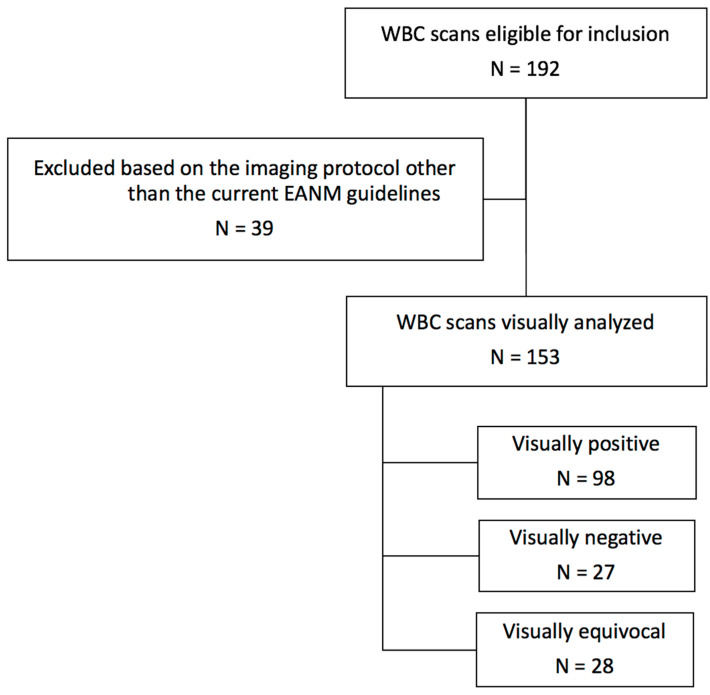
Flowchart of patient inclusion.

A total of 153 patients underwent WBC scintigraphy in accordance with the current EANM guidelines for suspected FRI between February 2012 and January 2017. Patient inclusion is visualized in Figure 2. Based on the criteria in the EANM guidelines, a total of 28 out of the 153 (18%) scans were labeled as equivocal based on visual assessment and consequentially analyzed using the semiquantitative method. Irrespective of the scan results, ten out of these 28 cases were ultimately diagnosed with FRI, all based on positive intraoperative cultures. Sixteen cases involved open fractures. Three cases involved the humerus, three involved the radius/ulna, nine involved the femur, 11 involved the tibia and two involved the foot. Indications for WBC scintigraphy were mal- or non-union (55%), suggestive clinical signs of infection (30%), fistulae/sinus/wound breakdown (8%) and persistent pain or loss of function (7%).

### Diagnostic Performance in Equivocal Scans

The average calculated difference in leukocyte uptake between the early and late scans was −1.1% (this implies a decrease in leukocyte uptake of 1.1% between the early and the late scans) with a range of 0.69%. Dichotomization of the ratios using the increase cut-off of >0% or a ratio of >0% was classified as a positive scan result and yielded three TP, eight TN, 10 FP and seven FN scan results with respect to the reference standard. This corresponds to a sensitivity of 30.0% (95% CI, 7–65), a specificity of 45.0% (95% CI, 23–68) and a diagnostic accuracy of 40.0% (95% CI, 22–59) of semiquantitative analysis of equivocal WBC scintigraphy.

When using the > 10% increase in ratio between the early and late scans as a cut-off point, two TP, 14 TN, three FP and nine FN results were found, resulting in a sensitivity of 18.2% (95% CI, 2–52), a specificity of 82.4% (95% CI, 57–96) and a diagnostic accuracy of 66.3% (95% CI, 46–83). When using the > 20% cut-off point, 0 TP, 16 TN, two FP and 10 FN results were found, resulting in a sensitivity of 0.0% (95% CI, 0–31), a specificity of 88.9% (95% CI, 65–99) and a diagnostic accuracy of 66.7% (95% CI, 46–83).

**Figure 3:**
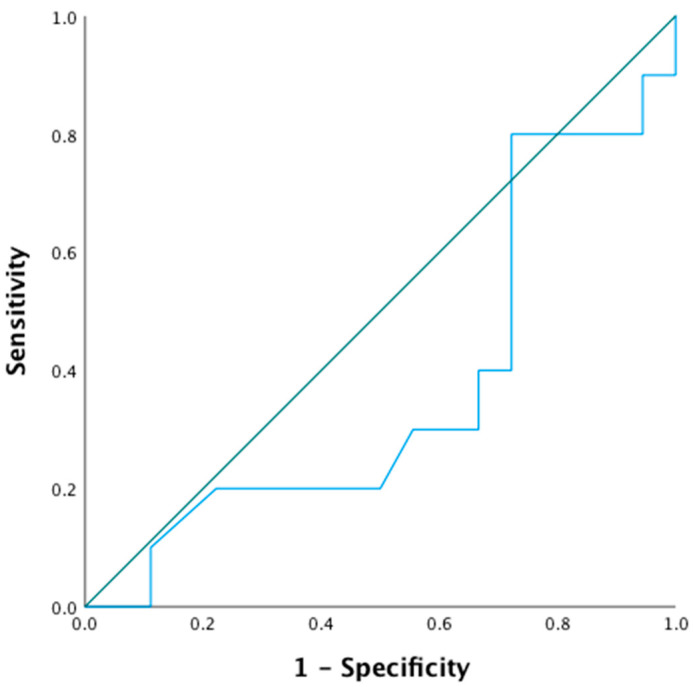
ROC curve of difference in the L/R ratio between the early and late scans, indicating poor discriminative ability.

When calculating the AUROC with the difference in leukocyte uptake between the early and late scans as a continuous variable, an AUROC of 0.37 (0.15–0.59) was found, indicating that semiquantitative analysis of visually equivocal WBC scans for FRI has poor accuracy regardless of the increase over the cut-off timepoint. In general, an AUC of 0.7 to 0.8 is considered acceptable, 0.8 to 0.9 is considered excellent and more than 0.9 is considered outstanding [12]. The ROC curve is displayed in Figure 3.

## 4. Discussion

The aim of this study was to assess the diagnostic value of semiquantitative analysis of visually equivocal WBC scintigraphy scans in patients with suspected FRI. The results demonstrate that semiquantitative analysis has poor diagnostic accuracy for FRI in equivocal WBC scans. Therefore, this method is insufficient for supporting clinical decisions in FRI treatment.

Literature on the value of semiquantitative analysis of WBC scintigraphy in musculoskeletal infections is limited. We identified only two studies in which the diagnostic value of semiquantitative analysis was reported. In 2004, Pelosi et al. assessed its value in patients with prosthetic joint infections (PJI) [10]. Seventy-eight patients with suspected infections of hip and knee prosthesis were included in this study and a triple timepoint imaging protocol was used: 50 min, 4 h and 24 h after the injection of 740 MBq [99m]Tc-HMPAO-labeled autologous leukocytes. The reference standard for PJI consisted of positive intraoperative cultures or, in non-operative cases, at least absence of PJI at one-year follow-up. The authors report a 95.7% sensitivity, 95.9% specificity and a diagnostic accuracy of 95.8% when using the semiquantitative method. They did not specify the criteria to consider an L/R ratio positive. Furthermore, a sub-analysis was performed for cases in which the visual analysis was ‘ambiguous’ (e.g., equivocal), which showed a decrease in sensitivity at 88%, with a specificity of 100% and a diagnostic accuracy of 96.2%. In 2013, Glaudemans et al. proposed an imaging and interpretation protocol for WBC scintigraphy in musculoskeletal infections [7]. This study encompassed multiple types of musculoskeletal infections including PJI, bacterial osteomyelitis, FRI, infections of the neuropathic (diabetic) foot, vertebral osteomyelitis and general soft tissue infections. The accuracy of the semiquantitative method was assessed and 82.0% sensitivity, 94.9% specificity and a diagnostic accuracy of 91.9% were found when using the semiquantitative method. In the sub-analysis of 49 patients with suspected infected osteosynthesis (i.e., FRI), they reported 100% sensitivity, 97.4% specificity and a diagnostic accuracy of 98%. A separate analysis of visual equivocal scans was not performed in this study.

The different results between these two previous studies and this study may be explained by differences in patient populations. Important differences exist between PJI and FRI, including the presence of a fracture in FRI, with different stages of callus formation, varying locations and accompanying soft tissue injuries. These may account for the higher diagnostic accuracy in the more homogeneous PJI population when compared to the more heterogeneous FRI population. Furthermore, inherent to the semiquantitative analysis method is the subjective nature of selecting the region of interest and the contralateral reference tissue. The EANM guidelines advocate the use of contralateral bone marrow as the reference tissue in suspected osteomyelitis or FRI. However, since the majority of FRIs involve the distal tibia (which has a limited amount of bone marrow), this study used the mirrored contralateral bone as the reference point in these cases. Furthermore, no exact guidelines exist regarding the size of the selected ROI and the reference point. Differences in surface area of the ROI used may also account for differences between this study and the results found in other studies.

Since the results of this study conclude that there is no role for semiquantitative analysis of equivocal WBC scans in FRI, another method of differentiating these ambiguous cases is needed. This study found visually equivocal scan results in almost 20% of the cases. A false positive or false negative scan result may lead to unnecessary surgery or delay in diagnosis. A recent study by Lemans et al. [3] found a high diagnostic accuracy of [18F]FDG-PET/CT in FRI, indicating that [18F]FDG-PET/CT may be a good alternative in patients with suspected FRI and visually equivocal WBC scintigraphy. Recently, the study protocol for the currently ongoing ‘Imaging techniques in patients with a suspected Fracture-related Infection’ (IFI) trial was published. This prospective multicenter study compares the diagnostic accuracy of MRI, WBC scintigraphy and FDG-PET/CT in patients with suspected FRI [13]. The results of this study will likely give more insight into alternative diagnostic imaging possibilities for patients with equivocal WBC scintigraphy.

## 5. Conclusions

The diagnostic accuracy of semiquantitative analysis of visually equivocal WBC scintigraphy in suspected FRI is insufficient for supporting clinical decisions in clinical practice.

## Figures and Tables

**Table 1:** Acquisition time correction for [99m]Tc decay.

Hours after the First Scan	Acquisition Time (s)	Acquisition Time (s)
	370–550 MBq	250–370 MBq
0	150	200
1	168	224
4	238	317
16	952	1269
16.5	1009	1345
17	1069	1425
17.5	1132	1510
18	1200	1600
18.5	1271	1695
19	1346	1795

## Data Availability

Data available on request due to restrictions e.g., privacy or ethical.
